# To Cover the Odor of Iodoform

**Published:** 1882-09

**Authors:** 


### To Cover the Odor of Iodoform.

Dr. Putz, of Graefrath, has tried all the recommended means for covering the odor of iodoform, and confines himself now exclusively to oil of mirbane or nitrobenzol, all the others having failed in his hands. Six drops of nitrobenzol are used for every gram of iodoform.—*Pharm. Zeit.; New Remedies.*

